# Adaptive Radiotherapy in the Era of Image Guidance and Artificial Intelligence: A Critical Review

**DOI:** 10.3390/cancers18142281

**Published:** 2026-07-16

**Authors:** Senthamizhchelvan Srinivasan, Christopher F. Njeh

**Affiliations:** Department of Radiation Oncology, School of Medicine, Indiana University, Indianapolis, IN 46202, USA; sensrini@iu.edu

**Keywords:** adaptive radiotherapy, image-guided radiotherapy, online adaptive radiotherapy, offline adaptive radiotherapy, MR-guided radiotherapy, cone-beam CT, artificial intelligence, dose accumulation

## Abstract

Adaptive radiotherapy (ART) transforms radiotherapy from a static treatment plan into a monitored, feedback-driven process that can respond to anatomic and biologic change. This critical narrative review synthesizes foundational physics literature, consensus reports, prospective studies, randomized trials, implementation reports, and ART-specific artificial-intelligence validation studies, with emphasis on image guidance, evidence quality, and clinical actionability. Further the review separates image guidance, reduced margins, gating, tracking, plan-of-the-day selection, offline replanning, and online reoptimization because these related interventions have different evidence bases. ART is most compelling when anatomic change is frequent, dosimetrically important, measurable, and not adequately managed by population margins or conventional image guidance. Quantitative evidence is strongest for dosimetric recovery, organ-at-risk sparing, workflow feasibility, and selected toxicity reduction; evidence for survival improvement remains limited and heterogeneous. Offline ART has mature roles for progressive or systematic change, whereas online MR-guided and cone-beam CT-guided workflows address daily anatomy through same-session contour review, plan selection or reoptimization, and rapid quality assurance. Artificial intelligence can accelerate segmentation, synthetic CT generation, planning, and quality assurance, but clinically meaningful validation requires geometric, dosimetric, time, failure-rate, bias, and post-update monitoring. ART should be deployed through explicit adaptation triggers, validated dose-accumulation methods, independent quality management, and prospective endpoints connecting adaptive decisions to toxicity, tumor control, workflow efficiency, cost, access, and equity.

## Simple Summary

Adaptive radiotherapy adjusts treatment when patient anatomy changes during radiotherapy. This review distinguishes routine image guidance and margin reduction from true plan adaptation and summarizes where evidence supports dose recovery, organ-at-risk sparing, workflow feasibility, or clinical benefit. Current evidence is strongest for dosimetric correction, toxicity reduction in selected margin-reduction settings, and safe delivery of high-dose treatment near mobile organs; proof of survival benefit remains limited. Artificial intelligence can shorten daily contouring and planning, but it also requires local validation, monitoring, and human oversight. The practical message is that adaptive radiotherapy should be used when a measurable change affects tumor coverage or normal-tissue safety and when the team can correct it through a validated and auditable pathway.

## 1. Introduction

Radiotherapy is indicated for a large proportion of patients with cancer and is commonly delivered with curative, adjuvant, definitive, or palliative intent. Population-based utilization models estimate that approximately half of patients will benefit from at least one course of radiotherapy during the cancer trajectory, although actual access varies across health systems and disease sites [1,2]. The central technical objective is straightforward but difficult: deliver a tumoricidal dose to the clinical target while keeping surrounding normal tissues below clinically meaningful tolerance.

The modern era of three-dimensional conformal radiotherapy, intensity-modulated radiotherapy, volumetric-modulated arc therapy, stereotactic body radiotherapy, and particle therapy has sharply improved the ability to sculpt dose. As conformality increases, however, treatment plans also become more vulnerable to geometric and dosimetric error; a highly conformal plan can fail if the anatomy treated today differs materially from the anatomy used for simulation and optimization [3,4]. Margins, robust optimization, immobilization, respiratory management, and image guidance are therefore not peripheral conveniences but core determinants of whether an intended plan is actually delivered.

Adaptive radiotherapy (ART) was proposed as a closed-loop treatment process in which observations acquired during the treatment course are used to modify patient setup, margins, dose prescription, or the treatment plan itself [5,6]. The concept reframed radiotherapy from a one-time planning exercise into an iterative process: image, measure, evaluate, adapt, verify, deliver, and learn. In its earliest implementations, ART relied on portal imaging and offline reassessment. Contemporary ART uses repeated volumetric imaging, deformable registration, automated segmentation, rapid optimization, and online quality assurance to decide whether the plan of the day is still appropriate.

Image-guided radiotherapy (IGRT) created the practical foundation for ART. Kilovoltage and megavoltage cone-beam computed tomography (CBCT), fan-beam CT-on-rails, ultrasound, implanted electromagnetic transponders, four-dimensional CT, and MR-guided systems have progressively increased the visibility of targets and organs at risk immediately before and, in selected systems, during irradiation [7,8]. These technologies reduce setup uncertainty but also reveal a deeper problem: correcting bony alignment or soft-tissue position does not necessarily correct organ deformation, tumor regression, weight loss, gas, bladder or rectal filling, or the changing relationship between target and organs at risk.

Motion management further complicates the picture. Respiratory motion, cardiac motion, gastrointestinal peristalsis, and voluntary or involuntary patient motion can occur during simulation, adaptation, and beam delivery. The AAPM Task Group 76 report established a framework for respiratory motion management, but intrafraction change remains a major determinant of whether an adaptive plan remains valid at the moment of treatment [9]. ART must therefore be distinguished from simple image guidance: ART changes the treatment strategy, whereas IGRT may only reposition the patient.

This review focuses on the practical and evidentiary distinction between offline and online ART, the disease sites in which adaptation is most mature, and the technologies that determine whether ART can be scaled safely. Prior reviews have described online ART workflows and technical considerations in detail [10,11]. The present review adds a more explicitly critical perspective by ranking evidence according to study design and endpoint maturity, summarizing quantitative clinical and workflow signals, distinguishing adaptation from adjacent technologies such as image guidance and gating, and proposing an implementation pathway that links adaptation triggers to quality assurance and documentation.

The clinical rationale for ART is therefore not that every new image should generate a new plan. Rather, ART should be considered when anatomy or biology changes enough to threaten target coverage, exceed a clinically relevant organ-at-risk constraint, or create a justified opportunity for margin reduction or dose escalation. This framing is important because many published ART reports demonstrate improved dose–volume histograms or faster workflows, whereas fewer demonstrate reduced toxicity, improved quality of life, local control, or survival. The novelty of this review is to make that hierarchy visible and to identify the operational safeguards needed before technical feasibility can be treated as clinical value.

This is a critical narrative review rather than a systematic review or meta-analysis. The literature was identified through targeted searches of PubMed/MEDLINE, Scopus, Web of Science, and Google Scholar updated through 30 June 2026, using combinations of the following terms: adaptive radiotherapy, adaptive radiation therapy, online adaptive, offline adaptive, MR-guided radiotherapy, MR-guided radiotherapy, CBCT-guided adaptive radiotherapy, dose accumulation, deformable image registration, auto-segmentation, synthetic CT, quality assurance, and artificial intelligence. Sources were prioritized if they were foundational physics articles, AAPM or consensus reports, randomized or prospective trials, multicenter studies, disease-site implementation reports, ART-specific AI validation studies, or studies with explicit quantitative workflow or clinical endpoints. Because the purpose was narrative synthesis, formal PRISMA screening, pooled effect estimation, and risk-of-bias scoring were not performed.

The term critical review is used here to indicate that studies were interpreted according to design hierarchy, sample size, single- versus multicenter experience, prospective versus retrospective methodology, dosimetric versus clinical endpoints, workflow reproducibility, duration of follow-up, and consistency with the broader literature. Randomized and prospective multicenter data were weighted more heavily for clinical conclusions; planning, phantom, and single-institution implementation studies were treated primarily as evidence of feasibility, dosimetric effect, or workflow performance. The quantitative tables emphasize representative studies with sample size, adaptive workflow, imaging platform, and key measured outcomes. Across the representative studies summarized in Table 1, Table 2 and Table 3, more than 790 patients or clinical cases and more than 1600 adaptive or simulated online fractions/sessions are represented; these data are heterogeneous and are not pooled as a meta-analysis.

Offline ART refers to adaptation performed between fractions, generally after physician and physicist review outside the treatment session. Online ART refers to same-session plan selection, contour modification, dose recalculation, or reoptimization while the patient remains on the treatment couch or in the treatment room. Real-time ART refers to approaches that modify or gate delivery during irradiation in response to continuous imaging, tracking, or physiologic feedback. MR-guided ART and CBCT-guided ART describe imaging platforms, not a specific adaptive decision. Improved image guidance, reduced margins, plan-of-the-day selection, online contour editing, online reoptimization, gating, and tracking are related but nonidentical interventions and should not be assumed to have identical clinical effects.

## 2. Sources of Uncertainty and Rationale for ART

The classical planning target volume framework assumes that uncertainty can be characterized, bounded, and covered by a margin. That approach remains indispensable, but it is least efficient when changes are systematic, progressive, nonrigid, or strongly patient-specific. Examples include tumor shrinkage and weight loss during head and neck chemoradiation, atelectasis resolution during lung radiotherapy, rectal distension during prostate radiotherapy, bladder and uterine motion during pelvic treatment, and stomach or bowel proximity during upper abdominal stereotactic radiotherapy.

Head and neck cancer illustrates the prototypical offline ART problem. Tumor regression, nodal shrinkage, salivary-gland displacement, and patient weight loss can alter both target coverage and organ-at-risk dose during a six- to seven-week course. Early serial CT studies quantified substantial volumetric and geometric changes during fractionated treatment, and replanning studies demonstrated dosimetric recovery in selected patients [12,13]. Prospective dosimetric data confirmed that adaptive replanning can improve delivered dose distributions, but clinical benefit is more difficult to prove; in the randomized ARTIX trial, weekly adaptive radiotherapy did not significantly improve the primary salivary-flow endpoint or most patient-reported outcomes compared with standard IMRT, although some imaging-based salivary parameters favored adaptation [14,15].

Thoracic radiotherapy highlights the interaction among tumor response, respiratory motion, and normal-tissue constraints. Lung tumors can regress rapidly, shift with atelectasis or effusion change, and move with respiration. Adaptive strategies have been proposed to preserve target coverage, reduce lung dose, and enable dose escalation in locally advanced non-small-cell lung cancer [16,17,18]. Prospective and clinical implementation studies suggest that adaptive replanning can maintain coverage and reduce lung dose in selected patients, but the magnitude of benefit depends on baseline anatomy, response pattern, imaging frequency, and the ability to distinguish true tumor extent from surrounding inflammatory or atelectatic change [19].

Pelvic radiotherapy demonstrates a different adaptive problem: the target may be stable or slowly changing, whereas adjacent hollow organs vary daily. In prostate cancer, rectal distension on the planning CT has been associated with increased biochemical and local failure, emphasizing that a single simulation anatomy can be misleading [20]. Real-time tracking with electromagnetic transponders reduced one component of uncertainty by monitoring prostate motion during delivery, and MRI-guided stereotactic body radiotherapy has shown how improved soft-tissue visualization and margin reduction may affect acute toxicity [21,22]. For cervix cancer, bladder, rectum, uterus, cervix, and nodal volumes can deform non-rigidly; plan-of-the-day and online adaptive approaches were developed because a single PTV expansion can be either insufficient on some days or unnecessarily toxic on others [23,24,25].

ART is therefore best understood as a response to uncertainty that is both observable and actionable. If the change cannot be measured reliably, adaptation may create false precision. If the change is measured but does not alter clinical objectives, adaptation may add complexity without value. If adaptation improves a dose–volume histogram but not the cumulative biologic dose delivered to relevant tissue, its clinical meaning is uncertain. The discipline of ART is the discipline of deciding which uncertainties deserve workflow complexity and which are already adequately managed by immobilization, margins, robust optimization, motion management, or conventional image guidance.

## 3. Principles of Adaptive Radiotherapy

**Figure 1:**
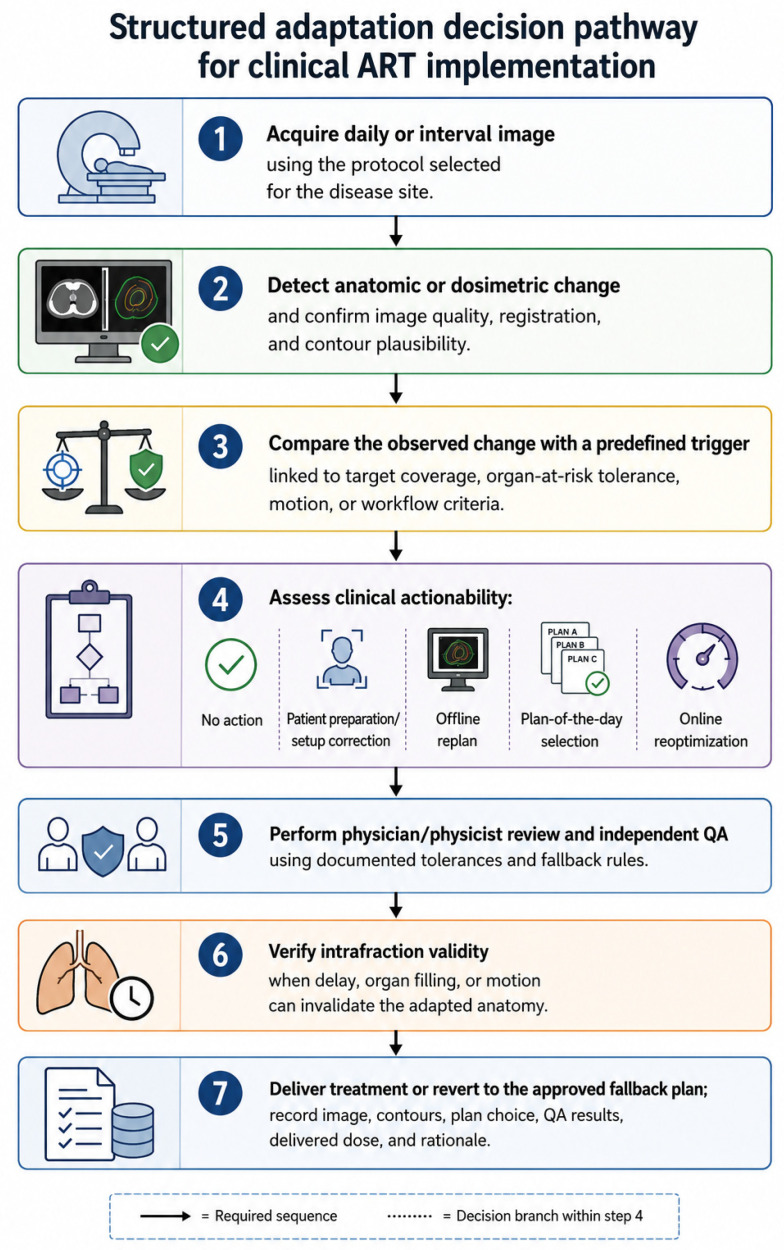
Structured adaptation decision pathway for clinical ART implementation.

A complete ART process has linked decisions: what to image, what to measure, what threshold should trigger intervention, what plan should be delivered, how the adapted plan should be verified, and how the delivered dose and decision should be recorded. Figure 1 shows a typical ART implementation decision pathway. The imaging decision determines the anatomic information available for adaptation. The measurement decision converts images into target, organ-at-risk, setup, motion, and dose metrics. The trigger decision determines whether the current plan remains acceptable. The planning decision selects a library plan, modifies contours, recalculates dose, or reoptimizes a plan. The verification and recording decisions determine whether the adapted plan is safe to deliver and whether cumulative dose information can inform future fractions.

Adaptation may be scheduled, triggered, or daily. Scheduled adaptation, such as repeat CT simulation after a specified number of fractions, is simple and operationally predictable but may miss early or late changes. Triggered adaptation uses observed criteria, such as weight loss, target shrinkage, organ-at-risk dose violation, setup trend, or physician concern. Daily adaptation is most personalized but requires the most automation, quality assurance, staffing, and treatment-room time. Table 1 compares offline and online ART across practical domains. Table 2 summarizes disease-site rationales, adaptive opportunities, and selected evidence.

**Table 1:** Practical comparison of adaptive radiotherapy workflows and quantitative performance signals.

Dimension	Offline or Scheduled ART	Online MR-Guided ART	Online CBCT-Guided ART	Practical Interpretation
Dominant uncertainty	Progressive or systematic change over days to weeks: weight loss, tumor regression, atelectasis, repeated setup trend.	Daily soft-tissue relationship immediately before treatment, especially near serial organs; cine MR can also support gating.	Daily anatomy visible on CBCT, especially pelvis, prostate, bladder, rectum, and selected head and neck or abdominal sites.	The workflow should match the dominant uncertainty; same-day adaptation is not automatically superior for slow systematic change.
Timing and QA window	Replanning occurs between fractions; conventional peer review, secondary dose calculation, and patient-specific QA can usually be completed before the adaptive fraction.	Same-session review, contour editing, plan adaptation, and online QA; treatment time and post-adaptation motion are important safety constraints.	Same-session contour review and adaptation using AI-assisted segmentation and template-driven optimization; early pelvic/prostate reports show adaptive procedure or total session times from approximately 17.6 min to 35 min, improving to a median total session time near 23 min in optimized prostate workflows [26,27,28].	Throughput and intrafraction validity should be measured prospectively and treated as quality endpoints.
Imaging and dose calculation	Repeat CT/4DCT, diagnostic MRI/PET fusion, or interval CBCT review; high-quality images may not represent anatomy at the next fraction.	Superior soft-tissue contrast, MR-based gating, and no additional ionizing imaging dose; synthetic CT or bulk-density methods require validation for dose calculation.	Broad access and integration with conventional linac workflows; CBCT image quality, scatter, truncation, and HU-to-density conversion require site-specific validation.	Image quality and electron-density accuracy determine whether the plan can be adapted safely, not merely whether daily anatomy can be visualized.
Best-supported evidence	Strongest for dosimetric recovery and selected prospective experiences; the ARTIX randomized trial in head and neck cancer showed no significant primary xerostomia or survival benefit despite imaging-based parotid improvement [15].	Randomized evidence exists for MR-guided prostate SBRT with margin reduction, but this is not a pure daily reoptimization trial; multicenter phase II evidence supports feasibility and safety signals for pancreatic SMART [22,29].	Evidence is expanding from implementation, prospective workflow, and dosimetric studies; multicenter randomized clinical endpoint data remain limited [26,27,28,30].	Claims should distinguish dosimetric, workflow, toxicity, local-control, and survival evidence.
Key quantitative signals	In prospective head and neck dosimetric data, one or two replans reduced parotid dose, but clinical translation remains uncertain [14,15]. In lung implementation, 27% of 233 patients required adaptation and mean lung dose decreased from 14.6 Gy to 12.6 Gy [19].	MIRAGE reported acute grade ≥2 GU toxicity of 24.4% with MR guidance versus 43.4% with CT guidance and acute grade ≥2 GI toxicity of 0.0% versus 10.5% [22]. In pancreatic SMART, adaptation was used in 93.1% of delivered fractions and no acute grade ≥3 GI toxicity was definitely attributed to SMART [29].	Sibolt et al. reported that >75% of AI segmentations required no or minor editing and adapted plans were superior in 88% of simulated cases; Byrne et al. reported no or minor influencer-contour edits in 92% of prostate fractions; optimized prostate workflows achieved ≤30 min total session time in 93% of sessions [26,28,30].	The strongest current quantitative support is for organ-at-risk sparing, toxicity reduction in selected margin-reduction settings, workflow feasibility, and safe high-dose delivery near mobile organs.
Primary limitations	May lag behind daily anatomy; benefit depends on trigger timing and whether the adapted plan remains valid for subsequent fractions.	Longer on-couch time, synthetic CT uncertainty, specialized staffing, MR contraindications, and post-adaptation motion.	CBCT soft-tissue contrast and auto-contour reliability vary by site; daily adaptation can normalize time pressure if stopping rules are weak.	Fallback plans, stopping rules, and audit trails are safety-critical for all workflows.

Abbreviations: ART = adaptive radiotherapy; CBCT = cone-beam computed tomography; CT = computed tomography; GI = gastrointestinal; GU = genitourinary; MR = magnetic resonance; MRI = magnetic resonance imaging; QA = quality assurance; SBRT = stereotactic body radiotherapy.

**Table 2:** Representative quantitative evidence by disease site, adaptive workflow, imaging platform, and evidence level.

Disease Site/Study and Evidence Level	Sample Size, Workflow, and Imaging	Key Quantitative Result	Critical Interpretation
Head and neck dosimetry: Schwartz et al.; prospective dosimetric clinical trial [14]	22 patients; all received one offline replan and 8 received two replans using daily CT-guided setup and deformable dose mapping.	One replan reduced contralateral parotid mean dose by 0.6 Gy and ipsilateral parotid mean dose by 1.3 Gy versus IGRT alone; two replans further reduced ipsilateral parotid dose by 4.1 Gy.	Supports dosimetric benefit, but was not powered to prove toxicity, quality-of-life, or survival improvement.
Head and neck clinical benefit: ARTIX; multicenter phase III randomized trial [15]	132 randomized, 131 analyzed, 11 French centers; weekly offline adaptive replanning versus standard IMRT for oropharyngeal cancer.	At 12 months, paraffin-stimulated salivary flow was 630 mg/min with ART versus 584 mg/min with IMRT (*p* = 0.64); parotid excretory function favored ART (48% versus 41%, *p* = 0.02); 2-year OS was 76.9% in both arms.	Key contradictory result: weekly ART did not improve the primary xerostomia endpoint or survival despite an imaging-based parotid signal.
Lung target coverage and lung dose: Moller et al.; large clinical implementation cohort [19]	233 monitored patients, 63 adapted (27%); daily CBCT soft-tissue matching with trigger-based repeat CT and replanning.	75% of adaptations corrected a decrease in tumor dose; surveillance CT showed decreased CTV coverage in only 2% of cases; mean lung dose decreased from 14.6 Gy to 12.6 Gy.	Demonstrates actionable trigger-based ART for lung, but tumor boundary, atelectasis, microscopic disease, and respiratory phase remain uncertainties.
Prostate margin reduction: MIRAGE; single-center phase III randomized trial [22]	156 randomized; MR-guided SBRT with 2 mm margin versus CT-guided SBRT with 4 mm margin.	Acute grade ≥2 GU toxicity was 24.4% with MR guidance versus 43.4% with CT guidance (*p* = 0.01). Acute grade ≥2 GI toxicity was 0.0% versus 10.5% (*p* = 0.003).	High-level evidence that MR guidance enabling margin reduction can reduce acute toxicity; not proof that daily online reoptimization itself improves outcomes.
Pancreas and upper abdomen: Parikh et al.; multicenter single-arm phase II SMART trial [29]	136 patients with borderline resectable or locally advanced pancreatic cancer; 0.35 T MR-guided on-table adaptive SBRT, 50 Gy in 5 fractions.	On-table adaptation was performed in 93.1% of fractions; no acute grade ≥3 GI toxicity was definitely attributed to SMART, although possible/probable grade ≥3 GI toxicity was 8.8%; 1-year OS from SMART was 65.0%.	Strong feasibility and safety signal for ablative adaptive therapy near bowel; single-arm design and postoperative events require cautious interpretation.
Pelvic CBCT-guided online ART feasibility: Sibolt et al.; simulation plus first clinical implementation [26]	39 pelvic cases, 100 simulated online adaptations, and first 5 treated patients; AI-driven CBCT-guided oART for bladder, rectum, anal, prostate, and sarcoma cases.	>75% of AI segmentations required no or minor editing; adapted plan was superior in 88% of cases; median adaptive procedure duration was 17.6 min; bladder PTV reduction was 42% with estimated bowel-cavity V45Gy reduction of 24–30%.	Supports feasibility and workflow/dosimetric effects, but clinical toxicity endpoints were not mature.
Prostate CBCT-guided online ART contours and plan quality: Byrne et al.; simulation and clinical implementation [30]	182 simulated online fractions and 184 delivered clinical fractions; Ethos CBCT-guided oART with AI influencer contours and adaptive planning.	11% of AI-generated influencer contours required no change and 81% required minor edits; adaptive plans were frequently selected and treatment times were clinically feasible.	Shows that AI-assisted contours can reduce workload, but manual review remains necessary and results are site- and platform-specific.
CBCT-guided online ART program scale: Stanley et al.; first-year implementation roadmap [27]	>1000 adaptive fractions in first clinical year; kV-CBCT online ART with institutional process mapping and staged rollout.	Program required 12 months from project inception to first patient and 12 months to deliver 1000 adaptive fractions; average overall treatment time was approximately 35 min and adaptive component approximately 20 min.	Demonstrates scalability but also quantifies staffing, training, and treatment-room opportunity cost.
Optimized prostate CBCT-guided online ART workflow: Malygina et al.; single-center clinical experience [28]	69 prostate patients, 1366 oART sessions; Ethos CBCT-guided oART with workflow and imaging optimization.	Automated posterior rectal wall contouring reduced mean adaptation time from 16.0 to 10.5 min; HyperSight reduced mean total session time from 25.8 to 23.3 min; 93% of sessions were ≤30 min.	Shows learning-curve and workflow optimization effects; generalizability depends on disease site, imaging hardware, staffing, and protocol design.

Abbreviations: ART = adaptive radiotherapy; CBCT = cone-beam computed tomography; CTV = clinical target volume; GI = gastrointestinal; GU = genitourinary; Gy = gray; IMRT = intensity-modulated radiotherapy; MR = magnetic resonance; oART = online adaptive radiotherapy; OS = overall survival; PTV = planning target volume; SBRT = stereotactic body radiotherapy; SMART = stereotactic MR-guided adaptive radiotherapy.

Dose accumulation is conceptually central but technically fragile. Rigid registration can support translational corrections but cannot represent deformation of bowel, parotid glands, bladder, rectum, uterus, or tumor. Deformable image registration is therefore attractive for cumulative dose estimation, yet accumulated dose should not be treated as an exact measurement. Particular caution is required near sliding interfaces, gas pockets, disappearing or shrinking tumors, surgical cavities, large deformations, poor image quality, or changes in anatomic topology. The evidentiary threshold should be higher when accumulated dose is used for an immediate clinical decision than when it is used for retrospective evaluation or quality improvement. AAPM Task Group 132 emphasized commissioning, validation, and use-case-specific uncertainty assessment for registration and fusion, and those principles are particularly important when dose accumulation drives plan adaptation [31].

Quality management must be designed into ART rather than added after implementation. The adaptive workflow includes new failure modes: incorrect image selection, segmentation error, propagated-contour error, inappropriate target editing, misregistration, wrong plan selection, suboptimal reoptimization objective, delayed treatment after adaptation, intrafraction anatomy change, and inadequate independent dose verification. AAPM Task Group 100 provides a framework for prospective risk analysis, process mapping, failure-mode identification, and targeted quality management that is well suited to ART programs [32]. Table 3 lists validation and monitoring priorities.

**Table 3:** ART-specific AI, dose-calculation, and online-QA validation domains with quantitative endpoints.

Validation Domain	Quantitative Endpoints to Report	ART-Specific Examples	Critical Interpretation
Daily auto-segmentation	Dice similarity coefficient, surface distance, Hausdorff distance, contour-editing time, no/minor/major edit rate, clinically unacceptable output rate, and dosimetric effect of contour edits.	Sibolt et al. reported >75% no/minor edits in simulated pelvic oART [26]. Byrne et al. reported 11% no-change and 81% minor-edit influencer contours for prostate oART [30].	Geometric overlap alone is insufficient; clinical acceptability, edit time, and dose consequences determine whether auto-contours are safe for online decisions [33,34].
Synthetic CT or density assignment	Mean absolute HU or electron-density error, target/OAR dose difference, gamma pass rate, failure cases with gas, metal, truncation, contrast, or unusual anatomy.	SynthRAD2023 provided a multicenter benchmark of 1080 patients for MRI-to-CT and CBCT-to-CT synthesis and showed that image similarity does not necessarily correlate with dose accuracy [35].	Dose-based evaluation should be mandatory when synthetic CT is used for adaptive dose calculation.
Automated planning and adaptation selection	Adapted-plan selection frequency, plan-quality objective pass rate, target V95/D95 recovery, OAR maximum or mean-dose improvement, monitor units, modulation complexity, and fallback rate.	Pelvic CBCT-guided oART simulations reported adapted plans superior in 88% of cases [26]; pancreatic SMART used adaptation in 93.1% of delivered fractions [29].	A high adaptation rate is not automatically high value; adaptation should be triggered by clinically meaningful target or OAR metrics.
Online independent dose verification	Secondary dose-calculation pass/fail rate, 3D gamma pass rate, calculation time, number of overridden warnings, and discordance between TPS and independent algorithm.	Bessieres et al. analyzed 124 MR-linac PSQA results and found adapted-plan delivery accuracy generally preserved [36]. Xu et al. analyzed 208 Unity adaptive plans; mean ArcCHECK gamma pass rates exceeded 99% for ATP and ATS workflows, and independent calculation pass rates were approximately 98% [37].	Measurement-based QA cannot usually precede same-day delivery; commissioning, independent calculation, periodic sampling, and stopping rules must compensate.
Workflow efficiency and intrafraction validity	Total session time, adaptation time, time from image to beam-on, second-image displacement, patient movement, rate of fractions exceeding time target, and treatment cancellation or fallback rate.	Stanley et al. reported approximately 35 min average total treatment time in a first-year CBCT oART program [27]. Malygina et al. reported 93% of prostate sessions within 30 min after optimization [28].	Time savings matter because long adaptive sessions can invalidate anatomy and reduce access for other patients.
Model governance and drift	Performance by scanner/protocol/site/body habitus, update/version audit, out-of-distribution detection, incident reports, and periodic revalidation against physician-edited contours and delivered plans.	CLAIM provides a reporting mindset for AI in medical imaging, but ART requires local version control, edited-contour audits, and post-update validation [33].	Human oversight should focus on high-risk structures and endpoints, not on passive review of every voxel.

Abbreviations: AI = artificial intelligence; ART = adaptive radiotherapy; CBCT = cone-beam computed tomography; CLAIM = Checklist for Artificial Intelligence in Medical Imaging; CT = computed tomography; HU = Hounsfield unit; MR = magnetic resonance; OAR = organ at risk; PSQA = patient-specific quality assurance; QA = quality assurance; TPS = treatment planning system.

The appropriate adaptation threshold should be linked to clinical endpoints. A parotid mean dose change may matter in head and neck treatment; a bowel maximum dose constraint may matter in pancreatic SBRT; target undercoverage may matter in lung dose escalation; bladder or rectum displacement may matter in prostate and cervix treatment. ART protocols should therefore specify not only contouring and planning rules but also what clinical harm the adaptation is intended to prevent.

## 4. Offline Adaptive Radiotherapy

Offline ART is the most mature adaptive paradigm and remains essential even in centers with online platforms. It is well suited to progressive anatomic change, complex replanning, multidisciplinary review, and cases in which adaptation is needed only once or a few times during treatment. The patient is imaged during the course of treatment, the delivered or expected dose is evaluated, and a revised plan is generated for subsequent fractions. Because the patient is not on the couch during replanning, offline ART allows fuller physician review, dosimetrist and physicist involvement, peer review, secondary calculation, and, when needed, repeat simulation.

Head and neck cancer has historically been the leading offline ART use case. Scheduled midcourse resimulation or triggered replanning can address weight loss, immobilization-mask looseness, tumor regression, nodal changes, and organ-at-risk displacement. The literature supports the dosimetric feasibility of this strategy, particularly for parotid sparing and target coverage, but the randomized ARTIX results caution against assuming that every dosimetric advantage will produce a measurable functional outcome [12,13,14,15]. A rational head and neck ART program therefore needs patient-selection triggers such as substantial weight loss, visible target shrinkage, high baseline parotid dose, anatomy that places serial organs at risk near tolerance, mask-fit change, or repeated dose-accumulation warnings. The clinical claim should be framed as selective dosimetric recovery and potential toxicity reduction rather than proven survival improvement.

For lung cancer, offline ART can address tumor regression, atelectasis change, and evolving normal-lung exposure. Repeat CT or four-dimensional CT during treatment may reveal opportunities to reduce normal-tissue dose or escalate tumor dose. However, lung adaptation is vulnerable to uncertainty in tumor boundaries, respiratory phase, microscopic disease, and inflammatory change. Adaptive dose escalation should therefore be embedded in explicit protocols with robust imaging criteria, respiratory-motion assessment, and cumulative lung, esophagus, heart, and spinal-cord dose evaluation [16,17,18,19].

In pelvic radiotherapy, offline and plan-library approaches are often more practical than full online reoptimization. Plan-of-the-day strategies for cervix cancer use pretreatment variable-bladder imaging to build a library of plans corresponding to different uterus and cervix positions; the daily plan is selected according to the anatomy observed before treatment. This approach reduces reliance on a single large margin while acknowledging that bladder and bowel anatomy can vary substantially day to day [23,24,25]. Prostate radiotherapy may use offline review of rectal and bladder preparation, fiducial or soft-tissue localization trends, and repeated imaging to identify patients needing replanning, coaching, or modification of preparation protocols [20,21].

The strengths of offline ART are reliability, safety review, and scalability. Its limitations are temporal: the adapted plan is not necessarily the plan needed for the next fraction, and anatomic changes may occur between the repeat image and plan delivery. Offline ART is therefore optimal when the anatomy changes over days to weeks, when same-day adaptation is not necessary, or when the complexity of replanning outweighs the incremental benefit of treating the immediately observed anatomy.

## 5. Online Adaptive Radiotherapy

Online ART moves adaptation into the treatment session. The daily image is acquired, targets and organs at risk are reviewed or edited, dose is recalculated on the anatomy of the day, and either a library plan or newly optimized plan is selected and verified before treatment. This process directly addresses the limitation of offline ART: the anatomy used for adaptation is the anatomy immediately preceding treatment. It is most compelling when daily organ motion or deformation drives clinically meaningful target or organ-at-risk dose variation.

MR-guided radiotherapy accelerated online ART because MRI provides superior soft-tissue contrast compared with CBCT for many abdominal, pelvic, and thoracic targets. Dedicated MR-guided systems and MR-linacs demonstrated the feasibility of MR-guided planning, gating, and online adaptation, including the clinical proof of concept of high-field MRI-linac treatment [38,39,40]. The clinical value is not simply better images; it is the ability to use those images to reduce margins, protect nearby serial organs, verify anatomy immediately before high-dose treatment, and, when appropriate, gate treatment. These mechanisms should be reported separately because margin reduction, gating, and daily reoptimization are not interchangeable interventions.

Prostate SBRT is a leading example in which improved image guidance and adaptive principles converge. In the MIRAGE randomized clinical trial, MR-guided SBRT using a 2 mm margin reduced acute grade 2 or higher genitourinary toxicity compared with CT-guided SBRT using a 4 mm margin (24.4% versus 43.4%) and reduced acute grade 2 or higher gastrointestinal toxicity (0.0% versus 10.5%) [22]. MIRAGE was primarily a comparison of imaging-guided margin-reduction strategies rather than a pure daily reoptimization trial. It therefore provides high-level evidence that better daily anatomy management and smaller margins can affect patient toxicity in prostate SBRT, but it should not be generalized as proof that all forms of online ART improve clinical outcomes.

Upper abdominal stereotactic radiotherapy is one of the strongest rationales for online adaptation. Pancreatic tumors and oligometastatic abdominal lesions often lie near stomach, duodenum, small bowel, colon, liver, and kidney. A nonadaptive plan may be limited by the closest organ-at-risk configuration observed at simulation, whereas a same-day adaptive plan can prioritize target coverage when organs are safely distant and prioritize organ sparing when they are near tolerance. Early MR-guided adaptive planning work established feasibility, phase I data supported safety in abdominal tumors, and institutional series suggested that ablative five-fraction regimens could be delivered with online adaptation [41,42,43]. In a multi-institutional phase II trial of stereotactic MR-guided on-table adaptive radiotherapy for borderline resectable and locally advanced pancreatic cancer, 136 patients were treated with 50 Gy in five fractions, adaptation was used in 93.1% of fractions, and no acute grade 3 or higher gastrointestinal toxicity was definitely attributed to SMART; however, possible or probable grade 3 or higher gastrointestinal toxicity and postoperative events require caution when interpreting safety and efficacy [29].

CBCT-guided online ART has broadened access beyond MR-guided platforms. AI-assisted contouring, rapid dose calculation, and template-driven plan optimization have enabled daily adaptive treatment for pelvic, prostate, bladder, rectal, gynecologic, and other disease sites. Early implementation reports demonstrated feasibility of AI-driven CBCT-guided online adaptation in the pelvic region, including 39 pre-treatment pelvic cases, 100 simulated online adaptations, >75% AI segmentations requiring no or minor editing, adapted-plan superiority in 88% of cases, and a median adaptive procedure duration of 17.6 min for the first five treated patients [26]. Prostate experience reported 182 simulated fractions and 184 clinical fractions, with no or minor edits for most influencer contours, while first-year program data documented more than 1000 adaptive fractions and average total treatment time around 35 min [27,30]. More recent prostate experience involving 1366 sessions in 69 patients suggests that workflow optimization and improved imaging can reduce total session time to approximately 23 min and keep 93% of sessions within 30 min [28].

The limitations of online ART are predictable. Time on the couch increases the risk that anatomy changes after adaptation but before or during delivery. Physician, physicist, therapist, and dosimetrist roles may compress into a narrow time window. Automated contours may appear plausible but be wrong. Dose calculation and secondary checks must be rapid without becoming superficial. Online ART should therefore be implemented with disease-site-specific checklists, contour-review priorities, stopping rules, and clear authority for reverting to a scheduled nonadaptive plan when adaptation is unsafe or unnecessary.

## 6. Technologies Enabling ART and the Role of Artificial Intelligence

ART depends on a chain of technologies that must function together, and the chain should be validated by the clinical decision it supports. Imaging must be geometrically accurate and appropriately calibrated for dose calculation or synthetic CT generation. Segmentation must identify targets, organs at risk, and avoidance structures at the precision required by the adaptive endpoint. Registration must map anatomy and dose between time points without implying more certainty than the images support. Planning systems must rapidly generate deliverable plans that meet target and organ-at-risk objectives. Quality-assurance systems must verify machine deliverability, calculation accuracy, and consistency between the approved plan and the plan to be delivered within the time limits of the treatment session.

AI is attractive because it addresses several bottlenecks simultaneously, but its value in ART should be judged by measurable adaptive outcomes rather than by automation alone. Deep learning can support daily auto-segmentation, image synthesis, dose prediction, plan optimization, treatment-time prediction, online QA triage, and adaptive decision support [44,45,46]. In ART, the most immediate use is segmentation: if daily target and organ contours can be generated reliably and edited quickly, online adaptation becomes operationally feasible. The most consequential future use may be decision support: determining when adaptation is likely to matter clinically. Table 3 summarizes quantitative validation domains that should accompany ART-specific AI deployment.

AI also changes the safety problem. A model can fail silently when image quality, anatomy, scanner calibration, disease presentation, body habitus, surgical alteration, organ filling, or institutional contouring practice differs from the training distribution. A daily auto-contour that is wrong in a consistent direction could systematically bias all adaptive fractions. AI-generated synthetic CT could introduce dose-calculation error in regions of metal artifact, gas, contrast, truncation, or unusual tissue composition. Multi-domain evaluation is therefore essential: geometric metrics such as Dice similarity and surface distance should be combined with physician acceptability, contour-editing time, dosimetric consequences, failure-rate reporting, and out-of-distribution testing [33,34]. The SynthRAD2023 challenge, which used a multicenter dataset of 1080 patients for MRI-to-CT and CBCT-to-CT synthesis, further illustrates that image-similarity metrics do not necessarily predict dose accuracy; dose-based validation is required when synthetic images drive adaptive dose calculation [35].

Human oversight remains central. Automation should reduce repetitive tasks and expose relevant decisions, not obscure them. A practical online ART interface should highlight changes in target coverage, dose to serial organs, contour edits, optimization tradeoffs, cumulative-dose implications, and independent QA status. Reviewers should not be asked to inspect every voxel equally; the system should guide attention to structures and dose metrics that determine whether adaptation is justified. This is especially important in high-dose-per-fraction treatments, where one contouring, synthetic CT, or registration error can consume a large fraction of organ tolerance.

The most robust ART programs will treat AI as a validated medical device embedded in a clinical process rather than as an autonomous decision-maker. Model commissioning should include site-specific image sets, edge cases, physician-contoured reference data, acceptance criteria, failure-mode analysis, and periodic revalidation after software updates. Clinical records should preserve the daily image, propagated and edited contours, selected plan, verification results, delivered dose, and reasons for adaptation or nonadaptation. Without those records, quality improvement, incident learning, model-drift detection, and outcomes research are impossible.

## 7. Implementation, Safety, and Value

Implementing ART requires more than purchasing an adaptive platform. Programs should begin with a disease-site business case and clinical problem statement: what failure mode is being solved, how often it occurs, what patient population is affected, what endpoint should improve, and what resources are required. Candidate use cases can be prioritized by the frequency of actionable anatomic change, proximity of serial organs at risk, dose per fraction, expected benefit of margin reduction or dose escalation, and availability of trained staff.

A safe implementation pathway includes multidisciplinary protocol development, dry runs, end-to-end testing, staff credentialing, prospective risk analysis, and staged clinical rollout. Every disease-site protocol should define simulation requirements, image acquisition, contouring hierarchy, plan-library or reoptimization rules, dose constraints, adaptation triggers, online approval steps, independent check method, intrafraction verification, documentation, and fallback conditions. For online ART, the fallback plan is not an afterthought; it is a safety-critical component that allows treatment to proceed when adaptation is not needed or cannot be performed safely.

Staffing models vary. Some online workflows require the radiation oncologist and physicist to be present or immediately available for every adaptive fraction; others allow protocolized review by specially trained teams with escalation criteria. The correct model depends on disease site, fractionation, regulatory environment, institutional culture, and automation maturity. Overly burdensome staffing can make ART unsustainable, whereas overly permissive staffing can normalize unsafe automation. Implementation reports should therefore quantify treatment time, physician and physicist effort, therapist workload, fallback frequency, and near misses rather than assuming that efficiency improves automatically with experience [26,27,28,30].

Value assessment should include patient-centered and system-centered outcomes. For patients, relevant endpoints include toxicity, patient-reported symptoms, local control, survival, treatment interruptions, comfort, and anxiety associated with longer on-couch time. For clinics, relevant metrics include treatment-room utilization, staff effort, replan volume, physician and physicist time, imaging dose, plan-quality improvement, incidents and near misses, and opportunity cost. At present, the most mature evidence supports dose improvement, margin reduction in selected contexts, workflow feasibility, and selected toxicity signals more strongly than survival improvement. A daily adaptive fraction that improves a dose–volume histogram but delays treatment for other patients may or may not represent high-value care; such judgments require local measurement and prospective outcomes data.

Equity also matters, but the current literature should be interpreted as raising implementation considerations rather than proving that ART has worsened disparities. ART platforms can be expensive and can require specialized personnel. If adaptive care is offered only to patients treated at large academic centers or only to disease sites with favorable reimbursement, the technology could widen access gaps. Conversely, AI-assisted CBCT-guided ART may eventually democratize adaptation if it reduces dependence on scarce specialist time and makes soft-tissue-informed treatment available in more settings. Prospective registries should therefore capture not only dose and toxicity but also access, race and ethnicity where appropriate, geography, insurance, rurality, treatment completion, treatment time, and reasons adaptation was not offered.

## 8. Future Directions

The next stage of ART will likely move from anatomy-only adaptation toward biologically informed and response-adapted radiotherapy. Functional imaging, diffusion MRI, perfusion imaging, hypoxia imaging, positron emission tomography, and radiomics may eventually guide dose redistribution within a tumor, treatment intensification for resistant sub-volumes, or de-escalation for responding normal tissues. The concepts of multidimensional radiotherapy, dose painting, and radiomics have long suggested that images contain treatment-relevant biologic information, but prospective validation remains the central barrier [47,48,49].

Proton and heavy-particle therapy add another adaptive imperative. Particle dose distributions are sensitive to range uncertainty, tissue-density change, setup error, and motion; anatomic changes that are dosimetrically modest for photons can be substantial for protons. Range uncertainty and the sensitivity of intensity-modulated proton therapy to calculation uncertainty, inter-fraction change, and inter-field motion provide a strong rationale for robust evaluation and adaptive replanning in selected proton patients [50,51,52]. Adaptive particle therapy will require accurate daily stopping-power estimation, fast robust optimization, motion interplay assessment, and efficient quality assurance.

Real-time ART remains an aspirational but increasingly plausible goal. Continuous MRI, fluoroscopy, surface guidance, electromagnetic tracking, and machine-learning-based motion prediction could enable beam gating, tracking, or dynamic replanning during delivery. The challenge is not only speed; it is accountability. A real-time system must know when its input data are reliable, when a predicted target position is uncertain, and when delivery should pause. The more adaptation shifts from physician-observed decisions to automated control loops, the more transparent validation and fail-safe behavior become essential.

The evidence base must also mature. Future ART trials should be designed around clinically meaningful hypotheses rather than generic dosimetric improvement. Examples include margin reduction to reduce toxicity in prostate SBRT, adaptive sparing of bowel in pancreatic SBRT, parotid or pharyngeal constrictor sparing in selected head and neck patients, adaptive target reduction or boost in lung cancer, and plan-of-the-day strategies in cervix and bladder cancer. Trials should prespecify adaptation criteria, cumulative-dose methods, imaging schedules, patient-reported outcomes, toxicity definitions, local-control endpoints, health-economic measures, workflow metrics, and access measures. Direct comparisons should maintain strict distinctions among image guidance, margin reduction, gating, tracking, plan selection, and online reoptimization.

Finally, ART data should be structured for learning health systems. Each adaptive fraction generates images, contours, plans, dose distributions, edits, approvals, quality-assurance outputs, and clinical outcomes. If these data are stored in interoperable formats with appropriate governance, they can support model improvement, comparative effectiveness research, and quality benchmarking. If they remain siloed, ART will remain labor-intensive and evidence-limited despite technical sophistication.

## 9. Conclusions

Adaptive radiotherapy is a mature concept entering a new implementation phase. Offline ART remains valuable for progressive anatomic change and complex replanning; online ART enables same-day response to daily anatomy; real-time ART and biologic adaptation are emerging frontiers. The central theme from the literature is that adaptation is not inherently beneficial. It is beneficial when an anatomic or biologic change is measurable, clinically relevant, correctable within a safe workflow, and connected to a meaningful endpoint. Current evidence is stronger for dosimetric recovery, organ-at-risk sparing, workflow feasibility, and selected toxicity reduction than for survival improvement.

The future of ART will depend less on whether images can be acquired or plans can be reoptimized and more on whether radiation oncology can define the right patients, thresholds, endpoints, and safety systems. AI will be indispensable for scale, but validated human-centered workflows will remain indispensable for trust. ART should therefore be deployed as a rigorous clinical strategy: protocolized, quality-managed, outcomes-driven, equitable, and continuously audited.

## Data Availability

No new data were created or analyzed in this study. Data sharing is not applicable to this article.
